# SE-OnionNet: A Convolution Neural Network for Protein–Ligand Binding Affinity Prediction

**DOI:** 10.3389/fgene.2020.607824

**Published:** 2021-02-19

**Authors:** Shudong Wang, Dayan Liu, Mao Ding, Zhenzhen Du, Yue Zhong, Tao Song, Jinfu Zhu, Renteng Zhao

**Affiliations:** ^1^College of Computer Science and Technology, China University of Petroleum (East China), Qingdao, China; ^2^Department of Neurology Medicine, The Second Hospital, Cheeloo College of Medicine, Shandong University, Jinan, China; ^3^Department of Artificial Intelligence, Faculty of Computer Science, Polytechnical University of Madrid, Campus de Montegancedo, Madrid, Spain; ^4^School of Economics, Beijing Technology and Business University, Beijing, China; ^5^Trinity Earth Technology Co. Ltd, Beijing, China

**Keywords:** protein-ligand binding affinity, molecular docking, deep learning, convolutional neural network, drug repositioning

## Abstract

Deep learning methods, which can predict the binding affinity of a drug–target protein interaction, reduce the time and cost of drug discovery. In this study, we propose a novel deep convolutional neural network called SE-OnionNet, with two squeeze-and-excitation (SE) modules, to computationally predict the binding affinity of a protein–ligand complex. The OnionNet is used to extract a feature map from the three-dimensional structure of a protein–drug molecular complex. The SE module is added to the second and third convolutional layers to improve the non-linear expression of the network to improve model performance. Three different optimizers, stochastic gradient descent (SGD), Adam, and Adagrad, were also used to improve the performance of the model. A majority of protein–molecule complexes were used for training, and the comparative assessment of scoring functions (CASF-2016) was used as the benchmark. Experimental results show that our model performs better than OnionNet, Pafnucy, and AutoDock Vina. Finally, we chose the macrophage migration inhibitor factor (PDB ID: 6cbg) to test the stability and robustness of the model. We found that the prediction results were not affected by the docking position, and thus, our model is of acceptable robustness.

## Introduction

The binding affinity of small molecules to receptor proteins is the key to drug discovery and drug repositioning (David Hecht, 2009; Ru et al., 2020; Zeng et al., 2020a). Chemical prediction methods are often time-consuming and costly. The development of accurate prediction models for calculating binding affinity is imperative. The OnionNet model (Zheng et al., 2019) was proposed for predicting binding affinity using the three-dimensional structure of complexes. In the search for a favorable docking pose, a specific scoring function is used to estimate the binding affinity often with a low accuracy and a high false-positive rate. For example, data experiments have been conducted using the comparative assessment of scoring functions (CASF) (Li et al., 2014a,b). We have also previously tested the performance of AutoDock Vina on the CASF-2013 benchmark (Gaillard, 2018). Additionally, the molecular mechanics Poisson–Boltzmann surface area method (Rd et al., 2012) was developed to calculate the binding free energy. This method is computationally intensive and is generally superior to the docking scoring function (Shoichet, 2004).

It was found that the performance of machine learning methods for predicting binding affinity is heavily dependent on the way proteins and ligands are represented. In virtual screening methods, for example, the output is usually analyzed using a docking software to generate or manually extract features of the protein–ligand interaction. This is a laborious and complicated process, and it cannot be efficiently applied in machine learning methods, particularly for large-scale data (Lheureux et al., 2017).

Deep learning technology aims to minimize the time taken for the feature extraction process. The non-linear transformation of the original dataset can reveal the principles hidden in a large-scale dataset. Recently, deep learning technology has attracted the attention of academia and has become a viable option for pharmaceutical research. Dahl et al. developed a multitask deep learning model to predict the chemical structure of molecules, the pharmacophore of the active site, and drug levels toxic to the active site (Lv et al., 2019; Lin et al., 2020; Zeng et al., 2020b). Ramsundar et al. proposed a deep neural network model that efficiently predicts drug activity and structure (Wallach et al., 2015; Jain and Kumar, 2019; Zhao et al., 2019).

In this study, we propose a modified deep learning model, called SE-OnionNet, with two Squeeze-and-Excitation (SE) (Hu et al., 2017) modules to estimate the binding affinity of a protein–ligand complex. Specifically, the SE module is used to increase the non-linear expression ability of the network. We first extract the feature map from the three-dimensional structure of the complex. The local and non-local interactions between each pair of proteins and ligands are identified by dividing the contact characteristics between the protein and the ligand into different distance ranges. Then, the feature map is inputted into the network, and a predicted value is obtained as the output. We tested our SE-OnionNet using the scoring functions on PDBbind (v. 2018) and CSAF-2016 benchmark (Altae-Tran et al., 2017) and found that our model performs better than the classical OnionNet model. In addition, we compare our model with AutoDock Vina's ranking function (Oleg and Olson, 2009). We found that our model can predict significantly higher number of complexes than AutoDock Vina. Our model can also use predicted ligand structures, from a docking simulation, as its input, indicating its robustness.

## Materials and Methods

### The OnionNet Model

**Figure 1:**
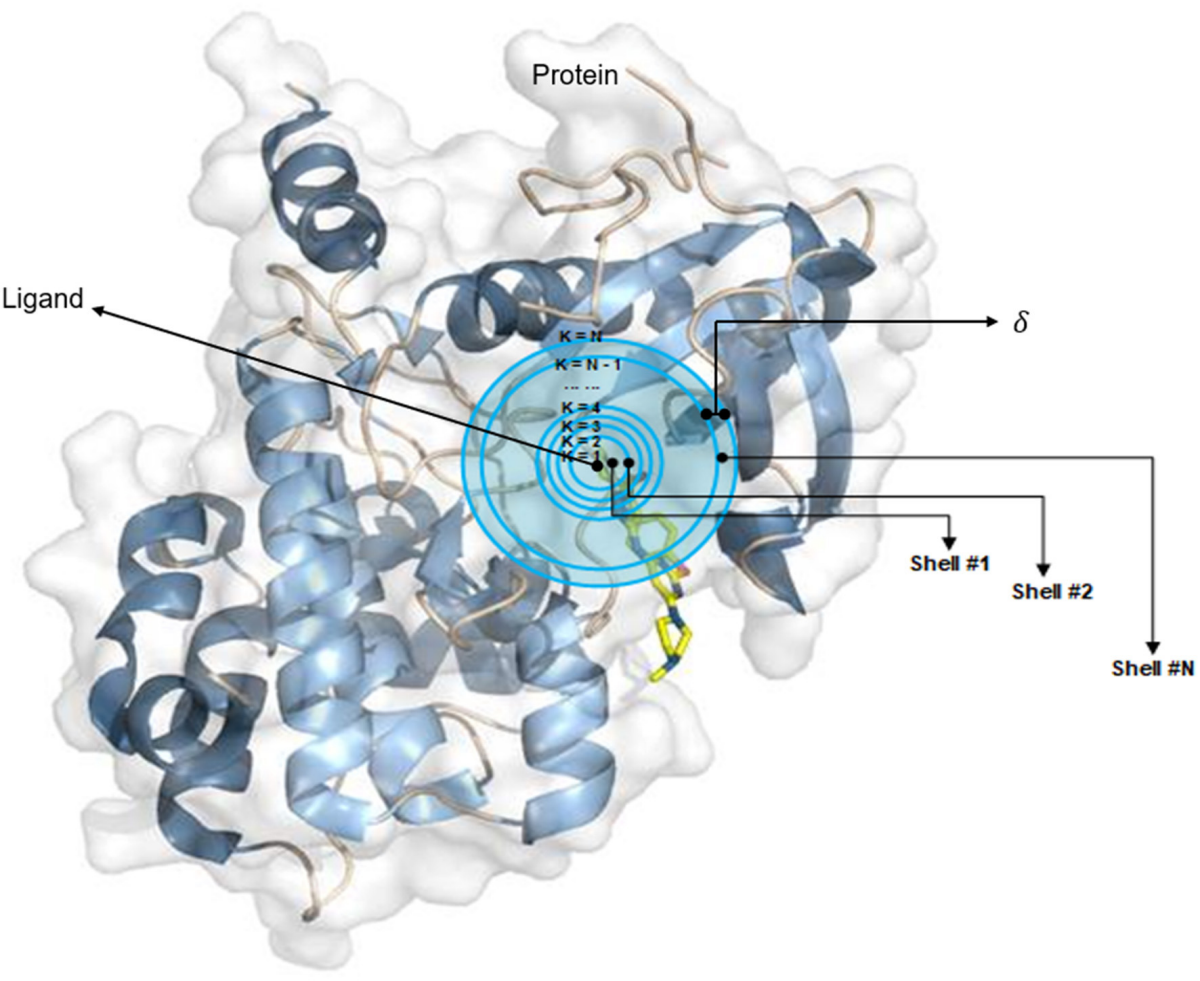
The definition of “shell.” The interaction between proteins and ligands is defined layer by layer in a three-dimensional space.

The OnionNet model was obtained by improving the characterization of protein–ligand complex data by Pafnucy, which used CASF-2013 as the benchmark. The three-dimensional structure of a protein–ligand complex is used as the input of the network. It defines each 1 Å as a three-dimensional box; extracts chemical information, centered on all ligand atoms in that box; and yields a high-dimensional (21 × 21 × 21 × 10) feature map. This is, then, inputted into the convolutional neural network model, which yields an affinity prediction ranking as the output. In addition, the OnionNet model defines “shell” as the boundary of each atom around a series of ligands. The “shell” is defined as the space between boundary *K* – 1 and *K*, with a thickness of δ (Figure 1). The *n*th shell is defined as the space between boundaries *k* = *n* – 1 and *k* = n, 1 ≤ *n* ≤ *N*. Intermolecular interactions between the ligand and the protein are expressed as the number of contacts between atoms in the *n*th shell.

They selected eight types of elements (*E*_*L*_), C, N, O, H, P, S, halogens, and all the remaining elements (ARE), to measure the types of contact between a ligand and the atoms in a protein. To maintain the generalization ability of the model, we define halogens to represent any one of the four elements F, Cl, Br, and I. For the *n*th shell, considering the different binding orientations of ligands and proteins, we used 64 features to represent the contact between the ligand and the protein.

(1) $$E_{L}=\left[\text{C},\text{N},\text{O},\text{H},\text{P},\text{S},\text{Halogen},\text{ARE}\right],$$

(2) $${\text{EC}}_{T_{S}T_{t}}=\sum_{r=1}^{R_{n,T_{s}}}\sum_{l=1}^{L_{T_{t}}}C_{r,l},\text{ while }T_{s}\in E_{L},T_{t}\in E_{L}$$

(3) $$c_{r,l}=\{\begin{array}{cc} 1, & \left(k-2\right)\delta +d_{0}\leq d_{r,l}<\left(k-1\right)\delta +d_{0}\\ 0, & d_{r,l}<\left(k-2\right)\delta +d_{0},d_{r,l}\geq \left(k-1\right)\delta +d_{0} \end{array},$$

For each element pair, EC_T_S_*T*_*t*__, the number of contacts is the sum of *C*_*r,l*_ between R_n,_T__s__ and *L*_*Tt*_, where *C*_*r,l*_, R_n,_T__s__, and *L*_*Tt*_ are the number of contacts, atoms in the protein, and atoms in the ligand, respectively. The *d*_*r,l*_ represents the distance between atom *r* and atom *l*, and the distance between the atom in the ligand to the nearest point of the boundary is defined as *d*_0_, if *d*_*r,l*_ is within (*k* – 2)δ + *d*_0_ ≤ *d*_*r,l*_ < (*k* – 1)δ + *d*_0_, then *r* and *l* is equal to 1; otherwise, it is equal to 0. In our study, we used the same values of *d*_0_ and δ as those used in OnionNet.

### The SE Module

**Figure 2:**
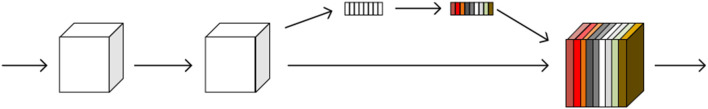
A squeeze-and-excitation block. The squeeze operation compresses the features along the spatial dimension and turns each two-dimensional feature channel into a real number. The excitation operation, which is similar to the gate mechanism in the recurrent neural network, generates weights for each feature channel. The reweight operation completes the recalibration of the original feature in the channel dimension.

The SE module is inspired by SENet, which was the champion of the ImageNet Large Scale Visual Recognition Challenge 2017. It allows for simple yet easy expansion within the existing network structure. The SENet network focuses on the relationships between channels, aiming to automatically learn the importance of different channel features. The SE module is shown in Figure 2.

The SE module performs a squeeze operation on the feature map to obtain the channel-level global features, and an excitation operation is performed on the global features to learn the relationship between the channels. The weight of the different channels is multiplied by the original feature map to obtain the final feature map. Essentially, the SE module performs an attention or a gating operation on the channel dimension. This mechanism can pay more attention to channel features, which have a large amount of information, while suppressing unimportant channel features. The SE module was embedded in our original network architecture.

### Our SE-OnionNet Model

**Figure 3:**
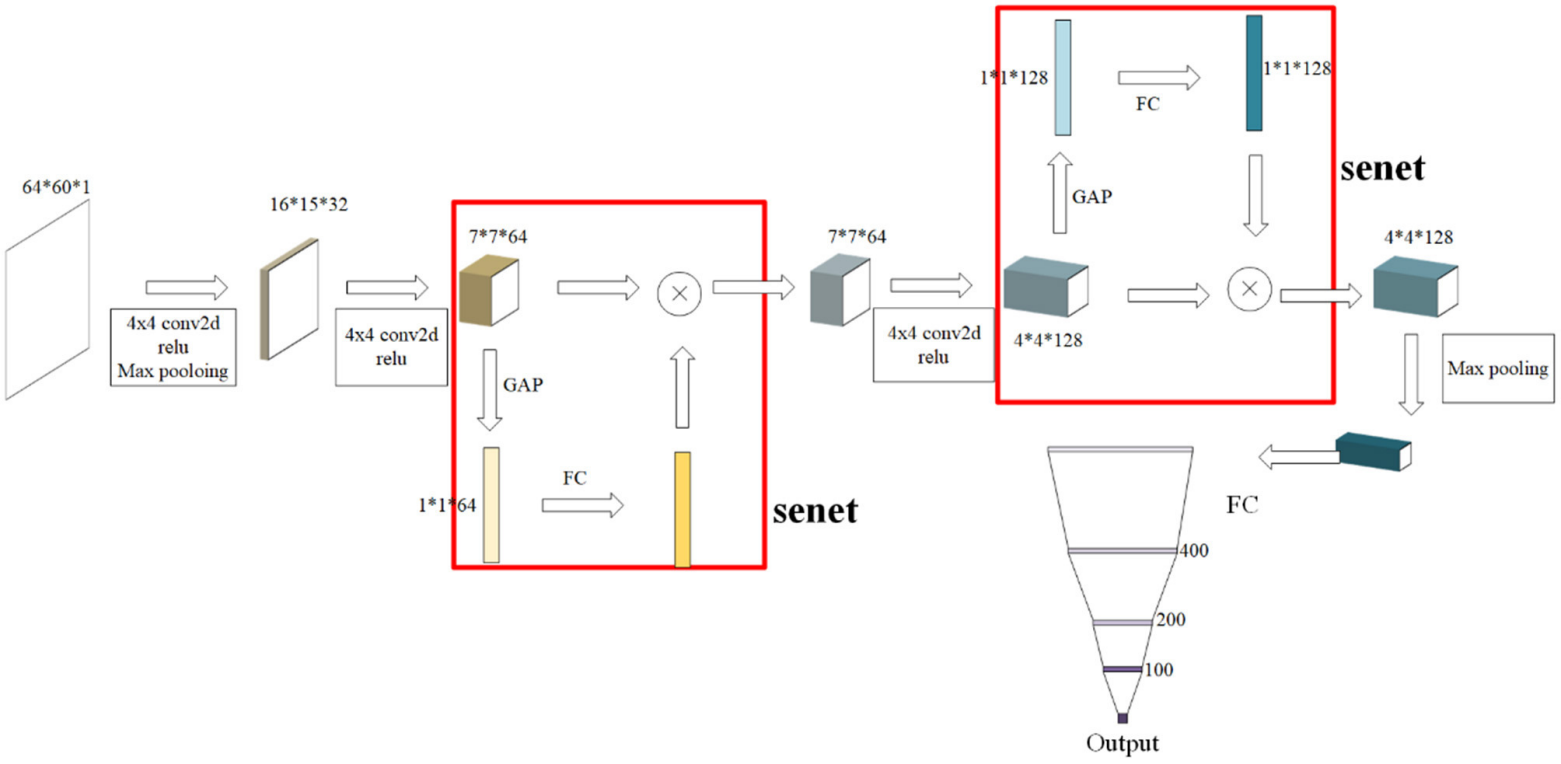
The structure of our model. It is composed of three layers of convolution, two SE blocks, and four layers of dense layer. SE blocks can not only effectively enhance the performance but also are computationally lightweight and impose only a slight increase in model complexity and computational burden.

In general, the SE-OnionNet model was designed by embedding SE modules, for their ability to perform attention operations, in the OnionNet network. Specifically, for each complex, two-dimensional information, as a feature map, is extracted from the three-dimensional structure. Then, the feature map is entered into a three-layer convolutional network to flatten and pass them onto the four fully connected layers with 400, 200, and 100 units, respectively. Finally, an output layer is generated with the predicted protein–ligand binding affinity score, p*K*_a_. The SE module is added to the second and third convolutional layers to improve the non-linear expression of the network. The structure of the SE-OnionNet model is shown in Figure 3.

We trained our model using the loss function in OnionNet, shown as follows:

(4) $$\begin{array}{l} \text{Loss}=\alpha \left(1-R\right)+\left(1-\alpha \right)\text{RMSE}, \end{array}$$

Where *R* and RMSE are the Pearson's correlation coefficient and root mean square error, respectively. α denotes an adjustable positive parameter that is <1. The value of α is set to be 0.8 in our model. The purpose of training is to obtain a higher *R* value and a lower RMSE value. We applied batch regularization to all layers except the last one, in order to avoid overfitting. For this, we tried many methods and finally chose to apply regularization between the convolutional layer and the dense layer. Adagrad was selected as the optimizer of SE-OnionNet after comparison with stochastic gradient descent (SGD) and Adam (Kingma and Ba, 2015).

### Evaluation Metrics

The Pearson's correlation coefficient (*R*), denoted in Equation (9), and the standard deviation (SD) are used to evaluate the performance of the model during the training process. The binding affinity, p*K*_a_, is expressed as the negative logarithm of *K*_*x*_, as follows:

(5) $$\text{p}K_{a}=-\log_{10}K_{x},$$

Where *K*_*x*_ represents the inhibition constant (*K*_i_), dissociation constant (*K*_d_), or semi-inhibitory concentration (IC_50_).

The accuracy of the model is evaluated by RMSE, calculated using Equation (6), to quantify the relative deviation between the predicted and experimentally measured values of p*K*_a_.

(6) $$\text{RMSE}=\sqrt{\frac{1}{N}\sum_{i=1}^{N}{\left(\text{p}K_{a_{\text{predict}}}-\text{p}K_{{\text{a}}_{\text{true}}}\right)}^{2}},$$

We also estimated the regression SD, calculated using the following equation:

(7) $$\begin{array}{l} \text{SD}=\sqrt{\frac{1}{N-1}\overset{N}{\underset{i=1}{\sum }}{\left(\left(a\;*\text{ p}K_{\text{a}}+b\right)-\text{p}K_{{\text{a}}_{\text{true}}}\right)}^{2},} \end{array}$$

where *a* and *b* are the slope and intercept of the linear regression line between the predicted p*K*_a_ and actual p*K*_a_ values.

The mean absolute error (MAE), calculated using Equation (8), is used to evaluate the prediction error.

(8) $$\begin{array}{l} \text{MAE}=\frac{1}{N}\sum |\text{p}K_{a_{\text{predict}}}-\text{p}K_{{\text{a}}_{\text{true}}}|, \end{array}$$

Finally, *R* calculated by Equation (9) is used to estimate the relationship between the predicted p*K*_a_ and actual p*K*_a_.

(9) $$\begin{array}{l} R=\frac{E\left[\left(\text{p}K_{{\text{a}}_{\text{predict}}}-\bar{\text{p}K_{{\text{a}}_{\text{predict}}}}\right)\left(\text{p}K_{{\text{a}}_{\text{true}}}-\bar{\text{p}K_{{\text{a}}_{\text{true}}}}\right)\right]}{\text{SD}\bar{\text{p}K_{{\text{a}}_{\text{predict}}}}\cdot \text{SD}\bar{\text{p}K_{{\text{a}}_{\text{true}}}}}, \end{array}$$

where $\text{SD}\bar{\text{p}K_{{\text{a}}_{\text{predict}}}}$ and $\text{SD}\bar{\text{p}K_{{\text{a}}_{\text{true}}}\;}$ are the standard deviations of the p*K*_a_ predicted by our network and the actual p*K*_a_, respectively.

### Datasets

**Figure 4:**
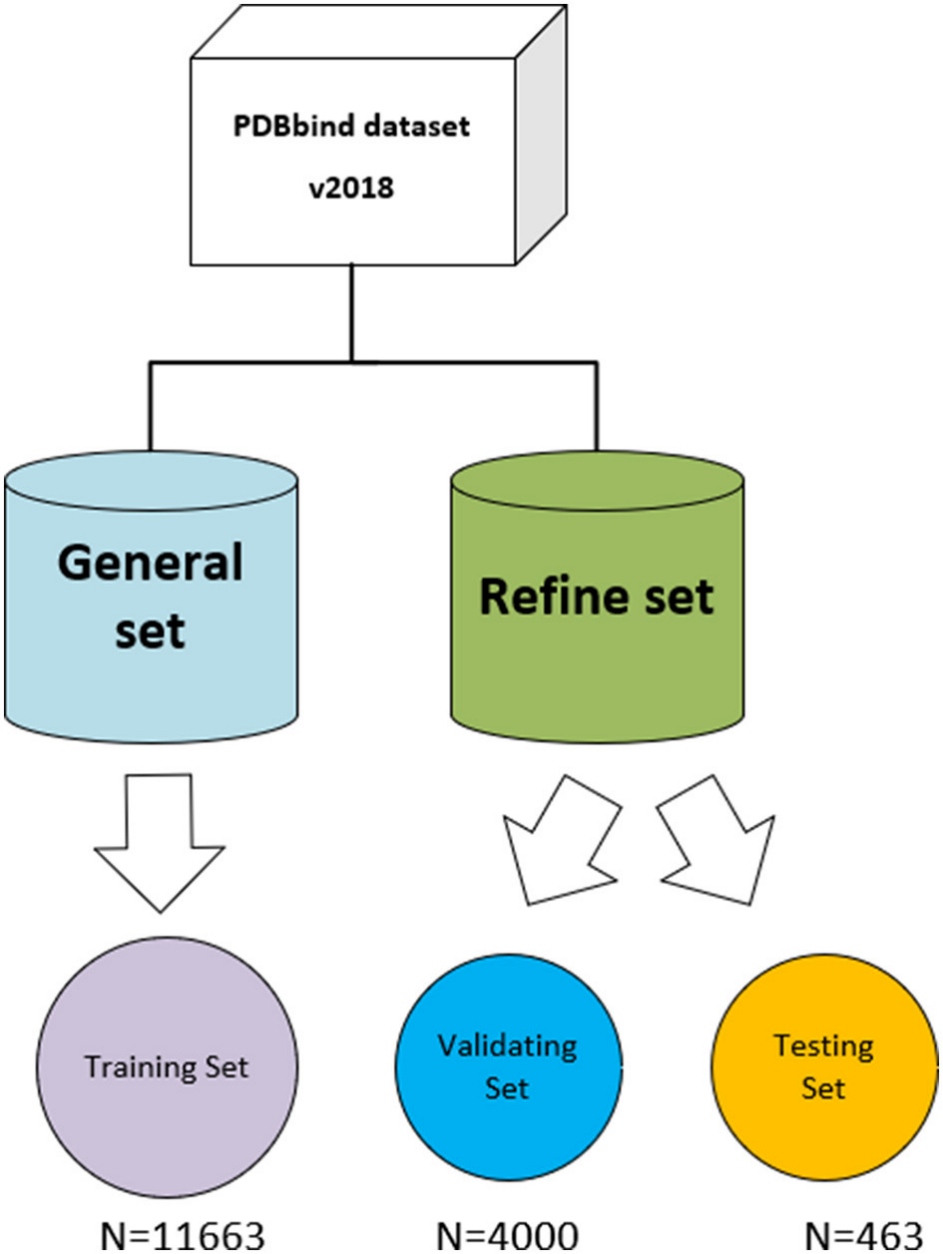
The datasets used in the model. The original PDBbind v.2018 dataset was filtered to retain only protein–ligand complexes with measured *K*_i_ or *K*_d_ binding affinity and divided into training set, validating set, and testing set.

The three-dimensional complexes used for the training and testing of our model are from the PDBbind database (v. 2018) (http://www.pdbbind.org.cn/). The dataset consists of both the general set, which includes 11,663 complexes, and the refined set, which includes 4,463 complexes, and the general set was used to train our model. We then randomly selected 4,000 complexes from the refined set for validation, and the rest were used as testing sets. The datasets used in the model are shown in Figure 4.

The CASF-2016 benchmark was selected to verify our model (Su et al., 2018). Compared with CASF-2013, CASF-2016 has improved in several aspects such as test set construction, evaluation method, and selection of scoring function. The CASF-2016 benchmark offers the following: (1) A larger and higher quality test set can be constructed; (2) there is an improved series of evaluation methods; and (3) 25 scoring functions can be tested for exemplary application.

## Results

### Performance Comparison With Different Optimizers

**Figure 5:**
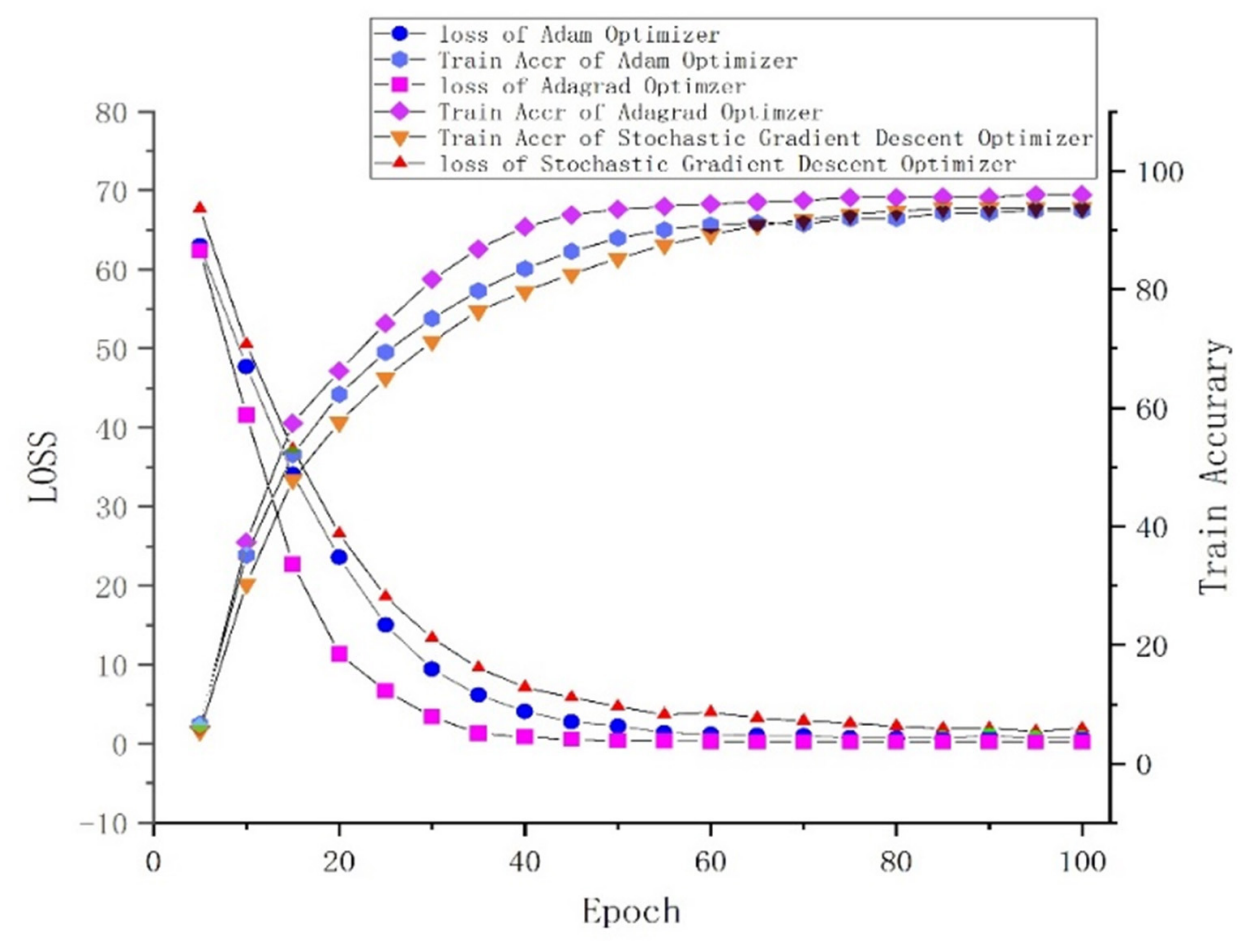
Loss and accuracy under different optimizers.

One of the central steps of the contemporary deep learning pipeline is to select an optimizer. Considering the sparsity of the feature map, we tried three popularly used optimizers: SGD, Adam (Kingma and Ba, 2015), and Adagrad (Duchi et al., 2011). The number of iterations for the optimization algorithm was set to 100, and the learning rate was set to 0.001. We found that Adagrad was the fastest optimizing algorithm with an accuracy higher than that of SGD and Adam. The loss and accuracy of the three optimizers, from 0 to 100 epoch(s), are shown in Figure 5.

### Performance of the SE-OnionNet Model

Our SE-OnionNet model performed well in the experiments conducted using the v. 2013 core and v. 2018 core datasets (see Table 1).

**Table 1:** Performance of SE-OnionNet.

**Dataset**	**R**	**RMSE**	**MAE**	**SD**
Training set	0.990	0.256	0.152	0.198
Validating set	0.814	1.584	0.823	1.221
V2013 core set	0.812	1.692	1.323	1.423
V2018 core set	0.853	1.592	0.912	1.253

For each complex in the dataset, the affinity was predicted and compared with the real value. The prediction accuracy of the model was evaluated based on the values of *R*, RMSE, SD, and MAE. The *R* values of our model were found to be 0.990, 0.814, 0.812, and 0.853 for the training set, validating set, and two testing sets, respectively, and these values are higher than those of the original model. The RMSE values calculated using Equation (6) were 1.584, 1.692, and 1.592 for the validating set and two testing sets, respectively, demonstrating that the p*K*_a_ predicted by our model is highly correlated with the actual p*K*_a_ value.

**Figure 6:**
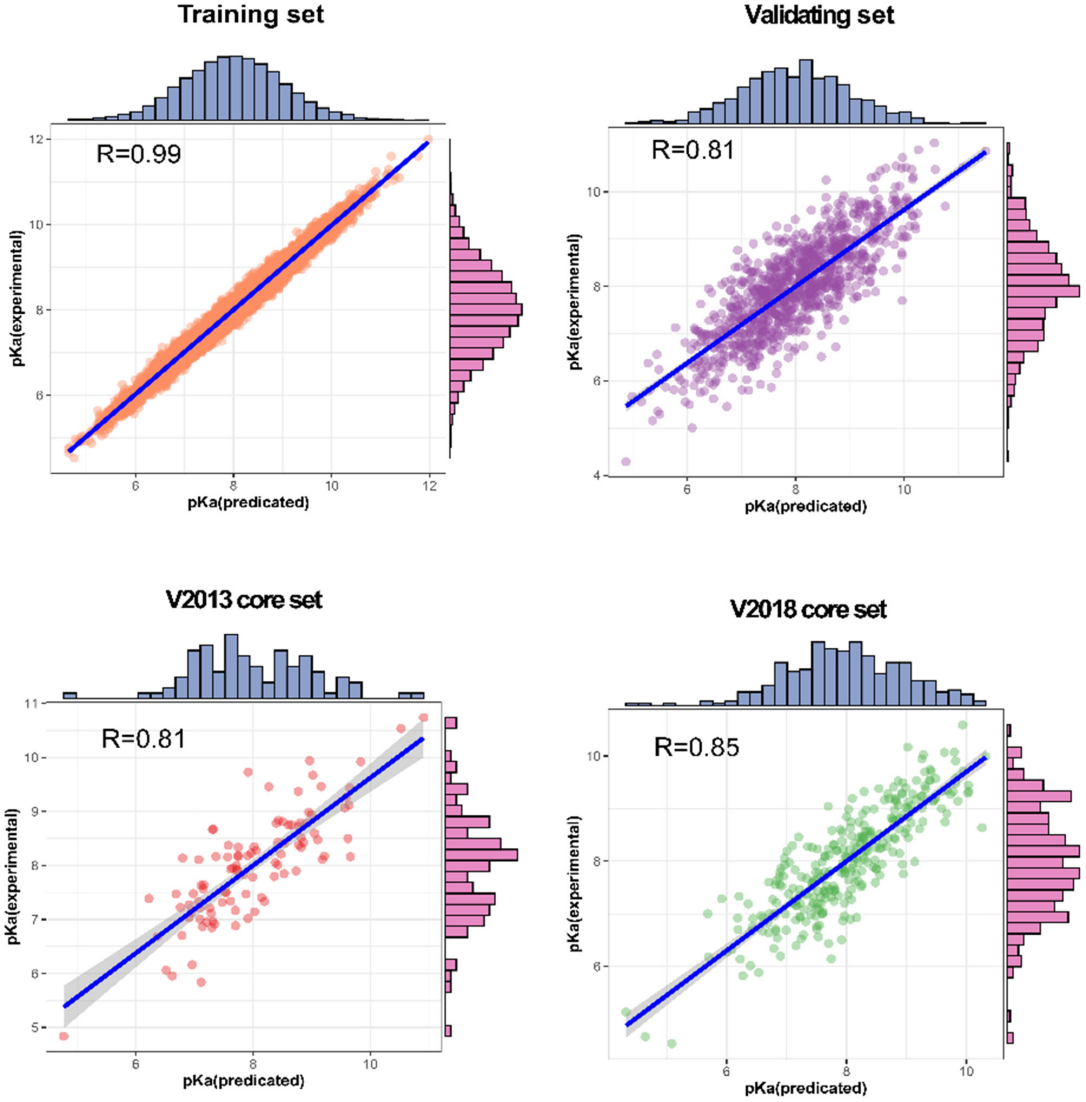
Predictions for two test sets (core sets from PDBbind v. 2013 and v. 2018): training set and validation set.

We also analyzed the correlation between predicted p*K*_a_ and measured p*K*_a_ of the different datasets using a scatter plot (Figure 6). As expected, the values were highly correlated not only in the training set but also in the validating and testing sets.

## Discussion

### Stability of SE-OnionNet

It is well-known that the stability of the deep learning model plays an important role. To test the model's stability, we compared the SD and *R* values of SE-OnionNet, OnionNet, Pafnucy, and AutoDock Vina using CASF-2016. The results are shown in Table 2. The SD and *R* values of our model are 1.20 and 0.83, while in the OnionNet, these are 1.26 and 0.82, respectively, indicating that our model is slightly better than OnionNet. Simultaneously, the indices SD and *R* of our model are better than those of Pafnucy and AutoDock Vina. Overall, our model based on deep learning performed better than other traditional scoring models.

**Table 2:** Performance comparison of different scoring functions.

**Scoring functions**	**SD**	***R***
SE-OnionNet	1.20	0.83
OnionNet	1.26	0.82
Pafnucy	1.37	0.78
AutoDock Vina	1.61	0.62

### Robustness of SE-OnionNet

**Figure 7:**
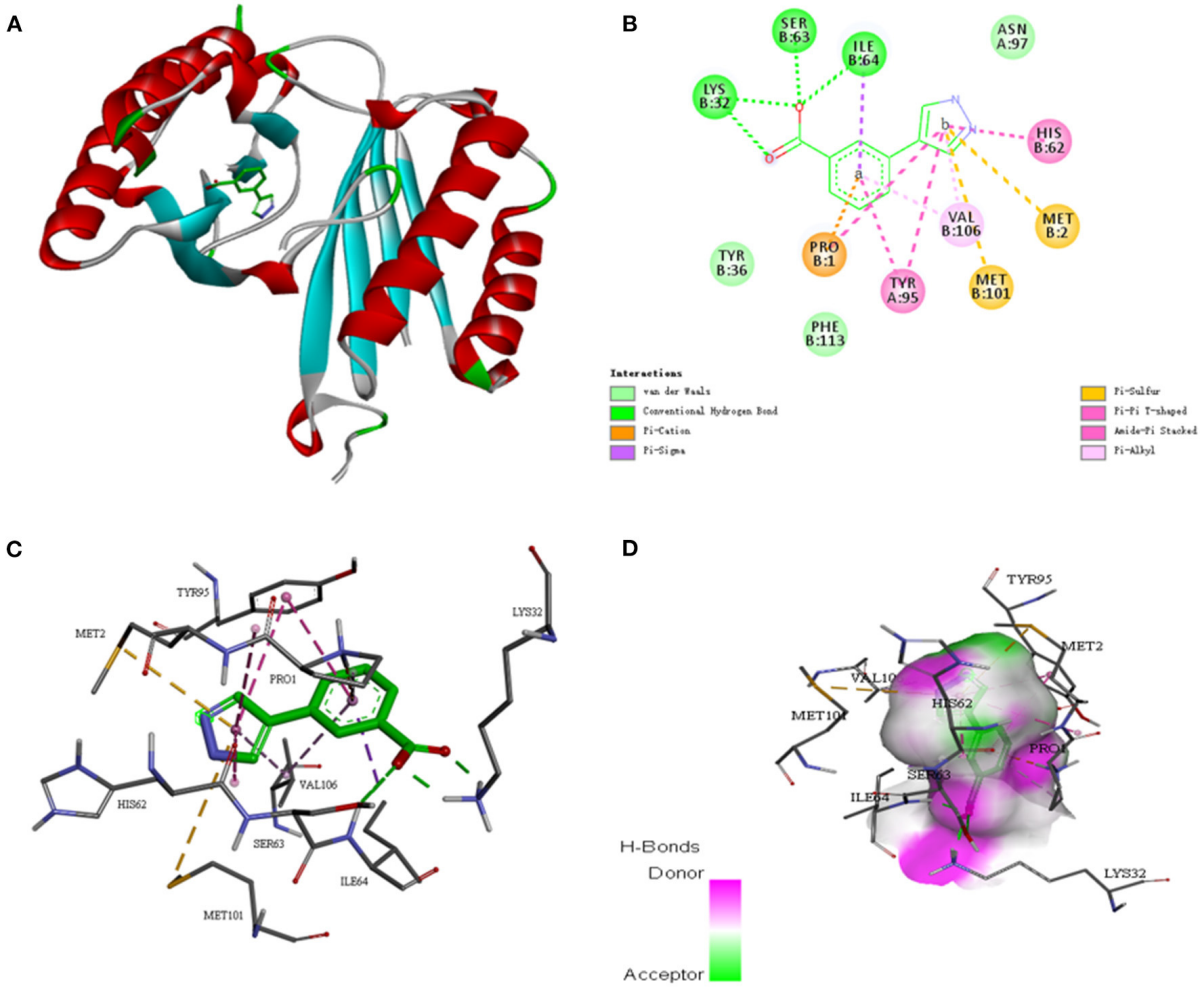
The interaction diagram between the macrophage migration inhibitor factor (PDB ID: 6cbg) and 3-(1H-pyrazol-4-yl)benzoic acid (EWG). **(A)** Molecular docking between the macrophage migration inhibitor factor (PDB ID: 6cbg) and 3-(1H-pyrazol-4-yl)benzoic acid (EWG). **(B)** Local two dimensional display of the interaction diagram between the macrophage migration inhibitor factor (PDB ID: 6cbg) and 3-(1H-pyrazol-4-yl)benzoic acid (EWG). **(C)** Three dimensional display of the interaction diagram between the macrophage migration inhibitor factor (PDB ID: 6cbg) and 3-(1H-pyrazol-4-yl)benzoic acid (EWG). **(D)** Hydrogen bond coloring display of the interaction diagram between the macrophage migration inhibitor factor (PDB ID: 6cbg) and 3-(1H-pyrazol-4-yl)benzoic acid (EWG).

To further investigate whether the SE-OnionNet model improved the robustness of the original model, we selected the macrophage migration inhibitor factor (PDB ID: 6cbg) and its ligand is 3-(1H-pyrazol-4-yl)benzoic acid (EWG) from the PDB refined dataset. The index p*K*_d_ (p*K*_a_ for our study) provided in the PDBbind database is 3.95. We first extracted the proto-ligand (EWG) from the protein and redocked it using AutoDock Vina (Figure 7). This was used as an input for our model to obtain the p*K*_a_ value. The predicted index p*K*_a_ of the complex, docked using AutoDock Vina, by our model was 5.645 and was not lower than the indexed p*K*_d_ provided in the PDBbind database.

## Conclusion

In this study, a modified deep learning model SE-OnionNet, with an attention mechanism to improve the performance of the model, is constructed. Based on the SENet model, we added SE modules to each of the two convolutional layers, except the first one, to improve the non-linear expression of the network and, thus, the performance of the model. We used three different optimizers, SGD, Adam, and Adagrad, to optimize the network, and finally, chose the superior Adagrad as our optimizer. Using CASF-2016, we found that the SE-OnionNet model outperforms the original model. Finally, for the purpose of testing the stability and robustness of the network, we chose the macrophage migration inhibitor factor (PDB ID: 6cbg) as an example. We found that our model is robust and the prediction results are not affected by docking orientation. We plan to add more modules to improve the performance of the model. Additionally, our study also motivates the formulation of innovative approaches to process the three-dimensional structure of a protein–ligand complex. Furthermore, it is worthwhile to use spiking neural networks [e.g., spiking neural P systems (Song et al., 2016, 2017, 2018, 2019a,b)] for drug discovery.

## Data Availability Statement

Publicly available datasets were analyzed in this study. This data can be found here: CASF-2016 (https://pubs.acs.org/doi/10.1021/acs.jcim.8b00545).

## Author Contributions

All authors listed have made a substantial, direct and intellectual contribution to the work, and approved it for publication.

## Conflict of Interest

RZ was employed by company Trinity Earth Technology Co. Ltd. The remaining authors declare that the research was conducted in the absence of any commercial or financial relationships that could be construed as a potential conflict of interest.
